# Anticoagulant Polyethylene Terephthalate Surface by Plasma-Mediated Fucoidan Immobilization

**DOI:** 10.3390/polym11050750

**Published:** 2019-04-28

**Authors:** Kadir Ozaltin, Marian Lehocky, Petr Humpolicek, Jana Pelkova, Antonio Di Martino, Ilkay Karakurt, Petr Saha

**Affiliations:** 1 Centre of Polymer Systems, Tomas Bata University in Zlín, Tr. Tomase Bati 5678, 76001 Zlín, Czech Republic; lehocky@post.cz (M.L.); humpolicek@utb.cz (P.H.); dimartino@utb.cz (A.D.M.); ykarakurt@utb.cz (I.K.); saha@utb.cz (P.S.); 2 Department of Hematology, Tomas Bata Regional Hospital, Havlickovo Nabrezi 2916, 76001 Zlin, Czech Republic; pelkova@utb.cz; 3 Faculty of Humanities, Tomas Bata University in Zlín, Stefanikova 5670, 76001 Zlín, Czech Republic

**Keywords:** polyethylene terephthalate, fucoidan, blood coagulation, anticoagulant, plasma treatment, surface coating

## Abstract

Biomaterial-based blood clot formation is one of the biggest drawbacks of blood-contacting devices. To avoid blood clot formation, their surface must be tailored to increase hemocompatibility. Most synthetic polymeric biomaterials are inert and lack bonding sites for chemical agents to bond or tailor to the surface. In this study, polyethylene terephthalate was subjected to direct current air plasma treatment to enhance its surface energy and to bring oxidative functional binding sites. Marine-sourced anticoagulant sulphated polysaccharide fucoidan from *Fucus vesiculosus* was then immobilized onto the treated polyethylene terephthalate (PET) surface at different pH values to optimize chemical bonding behavior and therefore anticoagulant performance. Surface properties of samples were monitored using the water contact angle; chemical analyses were performed by FTIR and X-ray photoelectron spectroscopy (XPS) and their anticoagulant activity was tested by means of prothrombin time, activated partial thromboplastin time and thrombin time. On each of the fucoidan-immobilized surfaces, anticoagulation activity was performed by extending the thrombin time threshold and their pH 5 counterpart performed the best result compared to others.

## 1. Introduction

Besides adequate mechanical performances of polymeric biomaterials, their surface properties (surface chemistry, hydrophilicity, energy and charge density) and their interactions with living tissue are equally essential to ensure biocompatibility. In the case of blood-contacting medical devices, such as vascular graft and stents, heart valves and intravascular catheters, regulating the blood–material interface is required to regulate hemocompatibility. The major drawback of such biomaterials (i.e., polyethylene terephthalate, polylactic acid, polycaprolactone and their nanofibers) is their insufficient hemocompatibility, which triggers thrombus formation on the material surface (namely, surface-induced thrombus formation) and may cause failure of the implanted material, vascular occlusion, heart attack and stroke [1,2,3,4,5,6,7]. 

The first response of the body to the implanted biomaterial is rapid protein adsorption within seconds, in accordance with the Vroman Effect, which leads to platelet adhesion and mediates thrombus formation [8,9,10,11,12]. This protein layer is recognizable by the integrin receptors of the cells; hence, protein adsorption is important for cellular interactions as well [13,14,15,16,17].

Platelet adhesion onto the protein layer is followed by platelet aggregation and activation of the intrinsic pathway of the blood coagulation cascade by blood protein factor XII (also called coagulation factor) to XIIa, fibrin network formation and finally complement system activation via erythrocytes and leukocytes interactions [18,19,20,21]. This activated blood coagulation cascade triggers thrombus formation on the biomaterial’s surface (surface-mediated thrombosis) [22]. 

Hence, reducing the protein adhesion at the beginning onto the biomaterial’s surface is the key point to prevent platelet adhesion, and therefore thrombus formation, for short- and long-term hemocompatibility of blood-contacting devices. 

Modification of the biomaterial’s surface can be considered for two approaches: first, tailoring the physical properties to change its surface topography, and therefore hydrophilicity and cellular interactions; second, chemical immobilization of a bioactive agent to obtain specific features according to the individual properties of the chemical agent. Immobilization of a bioactive agent onto a polymeric substrate is challenging due to the hydrophobic nature and lack of the bonding interfaces to create a covalent bond with the chemical agents for most of the polymeric materials. There are several methods to treat polymeric surfaces, such as wet chemistry, UV irradiation, corona discharge, flame treatment, ozone-induced treatment and plasma treatment [23,24,25,26,27,28]. Besides efficiency of the immobilization, desired bulk properties are equally important, especially for heat-sensitive and chemically unstable substrates. Therefore, plasma treatment is one of the best candidates for etching the surface to change its topography (therefore increasing its hydrophilicity and surface area) homogeneously and to introduce the beneficial functional hydroxyl, carboxyl and carbonyl groups on the surface for further chemical bonding with selected chemical agents. Due to the fact that plasma treatment is heat-free and its effect is limited to the top surface, the bulk properties remain unchanged.

Heparin is a well-known anticoagulant polysaccharide for avoiding blood thrombus, but its biggest drawbacks are hemorrhage and thrombocytopenia. Furthermore, it may cause animal-based infections since it is mostly obtained from animals [29,30,31,32,33].

**Figure 1:**
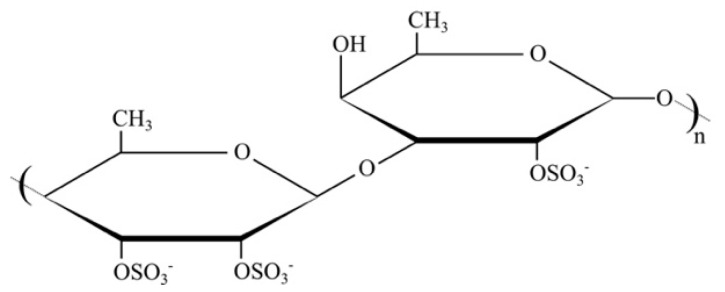
Chemical structure of fucoidan.

Fucoidan (Figure 1) is a marine-sourced sulphated polysaccharide, extracted from brown algae, with a better anticoagulant effect than that of anticoagulant heparin due to its action mechanism on the coagulation cascade by interacting with the natural thrombin inhibitors of antithrombin (AT III), heparin cofactor II (HCII), activated factor II (thrombin) and activated factor X [30,31,32,33,34,35,36,37,38]. Fucoidan has numerous biological activities besides its anticoagulant activity, such as anticancer, antitumor, antivirus and antiinflammation activities [39,40,41,42,43,44]. Its effect is related to monosaccharide composition, sulfation degree and pattern and molecular weight to regulate binding properties [32,33,34,44,45]. Furthermore, fucoidan does not cause hemorrhage as heparin does, and it has no risk of inflammation as animal-sourced heparin does, due to its marine origin [30,31,32]. 

Vesel et. al. studied the fucoidan from *Fucus vesiculosus* immobilization onto a polyethylene terephthalate (PET) surface after radio frequency (RF) plasma treatment at different pH values and demonstrated the immobilization performance by X-ray photoelectron spectroscopy. They found that the highest immobilization level occurred at pH 5, but anticoagulation activity was not carried out [46]. 

**Figure 2:**
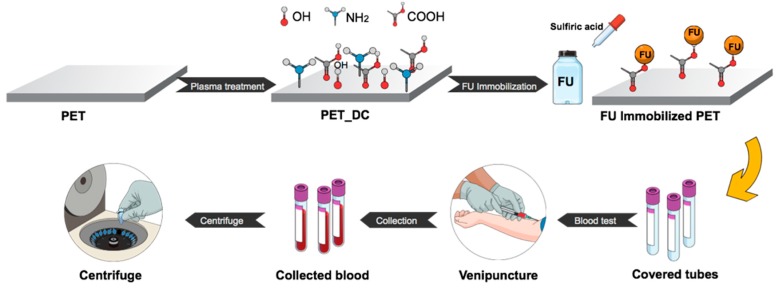
Experimental scheme.

In our previous study, fucoidan from *Fucus vesiculosus* was immobilized onto low density polyethylene at its natural pH value and it exhibited an anticoagulant activity slightly higher than the threshold [47]. In this study, polyethylene terephthalate (PET) was used as a substrate, due to its unique chemical and physical properties with highly crystalline structure, to create a functional anticoagulant surface using fucoidan from *Fucus vesiculosus* (FU). PET surfaces were activated by direct current (DC) plasma to form functional groups for FU immobilizations with pH levels of 3, 4, 5, 6 and 7 to compare its anticoagulation activity at various pH values, as it depicted in Figure 2. Surface characterizations were studied by wettability test, scanning electron microscopy, X-ray photoelectron spectroscopy and Fourier transform infrared spectroscopy. The anticoagulation activity of the samples was carried out for prothrombin time (PT), activated partial thromboplastin time (aPTT) and thrombin time (TT). 

## 2. Materials and Methods 

### 2.1. Materials and Preparation of Anticoagulant Surfaces

In this work, polyethylene terephthalate (PET) sheets with 70 × 30 × 0.1 mm dimensions were used as a polymeric substrate. All PET substrates were thoroughly cleaned with distilled water and dried at 30 °C for 24 h in an oven. PET substrates were exposed to direct current (DC) plasma at 50 W of reactor power and 40 kHz of frequency, generated by a PICO (Diener, Germany) plasma reactor under the chamber pressure of 50 Pa. As a discharge gas, air was used with 20 sccm flow rate, and both sides of PET sheets were treated for 60 s, hereafter referred to as PET_DC. Anticoagulant fucoidan from *Fucus vesiculosus* (FU) (Sigma Aldrich, St. Louis, MO, USA) solution was prepared as 1% (w/v) in distilled water and placed in the vials to change their pH. The total volume of the FU solutions in vials was 4 mL each. The pH values of the FU solutions of pH = 3, 4, 5, 6 and 7 were obtained by diluted H_2_SO_4_ addition. PET_DC samples were subsequently placed into each solution vial containing FU solution with pH = 3, 4, 5, 6 and 7 and placed in a rotational shaker for 24 hours at room temperature in order to immobilize anticoagulant fucoidan onto the functionalized surface of PET by plasma. Samples were labeled as FU3, FU4, FU5, FU6 and FU7, respectively. Finally, the samples were taken out of the solution vials, thoroughly washed with distilled water to eliminate non-immobilized fucoidan species and dried at room temperature for 24 h. Each sample was placed into a separated blood collection tube for further anticoagulation activity evaluation. 

### 2.2. Surface Wettability Evaluation

The wettability behavior of the samples to determine its surface hydrophilicity was studied by the Sessile drop method via SEE System (Advex Instruments, Brno, Czech Republic) equipped with a CCD camera. Distilled water was used as the testing liquid at 22 °C and 60% relative humidity. Ten separate droplets with a volume of 5 μL were placed onto each sample surface for 30 s to obtain the average water contact angle value (Q_w_). 

### 2.3. Surface Morphology Investigation by SEM 

Surface morphology of all samples was monitored by a NANOSEM 450 (FEI, Hillsboro, OR, USA) scanning electron microscope (SEM), equipped with a low vacuum detector and operated at 5 kV under 90 Pa pressure in a water vapor environment. Images were taken at the magnification of 10 k × with a spot size of 50 nm. 

### 2.4. Attenuated Total Reflectance Fourier Transform Infrared Spectroscopy (ATR-FTIR) Analysis

For surface chemistry examination, in order to compare the changes in chemical compositions of the studied samples, a Nicolet iS5 (Thermo Scientific, Grand Island, NY, USA) single beam Fourier transform infrared spectroscopy (FTIR) equipped with iD5 attenuated total reflectance (ATR) was used. Collected spectra were recorded between 400 and 4000 cm^−1^ with a resolution of 2 cm^−1^ and 64 scans using a ZnSe crystal at an incident angle of 45°.

### 2.5. X-Ray Photoelectron Spectroscopy (XPS) Analysis

To reveal the changes in chemical composition on the surfaces of the samples, X-ray photoelectron spectroscopy (XPS) analysis was carried out on a bioemulsifier film deposited on a glass slide using ESCALAB 200A, VG Scientific (East Grinstead, UK), with PISCES software for data acquisition and analysis. An achromatic Al (K_a_) X-ray source was operated at 15 kV (300 W), and the spectrometer was calibrated with reference to Ag 3d_5/2_ (368.27 eV) and operated in constant analyzer energy (CAE) mode with 20 eV pass energy. Data acquisition was performed with a pressure lower than 10^−6^ Pa. Gaussian–Lorentzian peak shape and Shirley type background subtraction were used for peak fitting of the spectral analysis.

### 2.6. Evaluation of Anticoagulation Activity

The blood was obtained by venous puncture from a healthy donor in accordance with the Helsinki Declaration, and placed into blood collection tubes (5 mL each) covered by prepared PET samples. The obtained human blood plasma was treated with 3.2% citric acid (109 mmol/L) and then centrifuged at room temperature, 3000 min^−1^, for 15 min. Anticoagulant activity was determined by means of prothrombin time (PT), thrombin time (TT) and activated partial thromboplastin time (aPTT) using a SYSMEX CA 1500 (Siemens, Munich, Germany) instrument. Each of the samples was examined three times. 

## 3. Results

### 3.1. Surface Wettability Behavior

Surface wettability results of studied samples, obtained by water contact angle, are given in Table 1. The water contact angle of 70.6° was monitored for the initial PET sample. This value of the water contact angle is referred to as a hydrophobic nature of the PET surface, which is not convenient for anticoagulant agent immobilizations. After DC plasma treatment (PET_DC), the water contact angle value sharply decreased to 22.09°, due to the introduced functional groups and the increased surface area by plasma, referring to the increased hydrophilicity and surface energy, which is convenient for further anticoagulant agent immobilizations. As can be seen in Table 1, FU-immobilized samples at various pH (samples of FU3 for pH 3; FU4 for pH 4; FU5 for pH 5; FU6 for pH 6 and FU7 for pH 7) showed almost the same water contact angle values within a range of 39.08° to 43.21°. Such an increase, compared to the plasma-treated sample, indicates the immobilization of FU onto PET_DC counterparts, as was demonstrated by SEM analysis. 

### 3.2. Surface Morphology 

**Figure 3:**
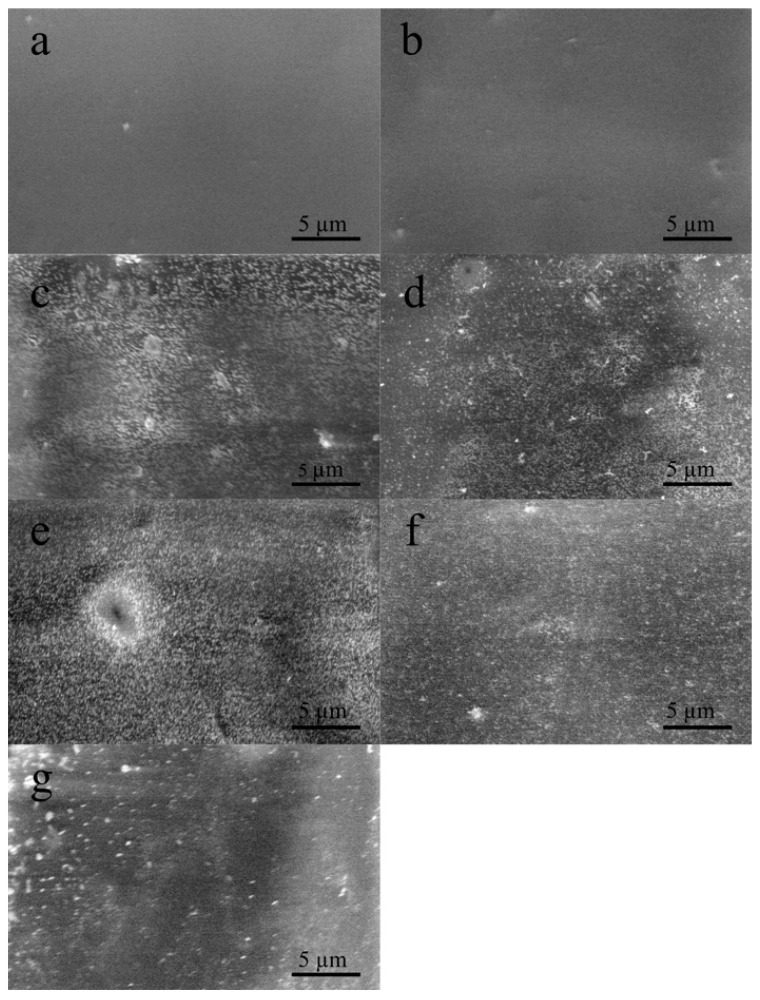
Surface morphology of fucoidan immobilized surfaces at various pH values: (**a**) PET; (**b**) PET_DC; (**c**) FU3; (**d**) FU4; (**e**) FU5; (**f**) FU6; (**g**) FU7.

The immobilization of fucoidan onto functionalized PET surface was investigated by scanning electron microscope, and the results are shown in Figure 3. As it is seen in Figure 3a, the reference PET surface is smooth without impurities; however, after DC plasma treatment it becomes relatively rough due to the increased surface area by plasma treatment (Figure 3b). The immobilization of fucoidan species on each sample was monitored, which revealed successful bonding to functional groups created by plasma. Nevertheless, the particle size and distribution of immobilized fucoidan species were different for each sample, due to the dissolution ability of fucoidan at different pH values. According to the SEM results, solubility and immobilization affinity of fucoidan seemed to be the best at pH 5 (Figure 3e) and pH 4 (Figure 3d). For both FU5 and FU4 samples, the particle size was smaller (this reduces the fraction of floating blood to decrease the chance of clustering blood particles such as fibrin and red blood cells, which trigger blood clot formation), and the distribution was more homogenous than that of the FU3 (Figure 3c), FU6 (Figure 3f) and FU7 (Figure 3g) counterparts. This was also confirmed by XPS results, showing that sulphur content was much higher for FU5 and FU4 compared to others. Furthermore, as expected, their anticoagulation activity was better, which is discussed later on.

### 3.3. Surface Chemical Anaylsis by ATR-FTIR 

**Figure 4:**
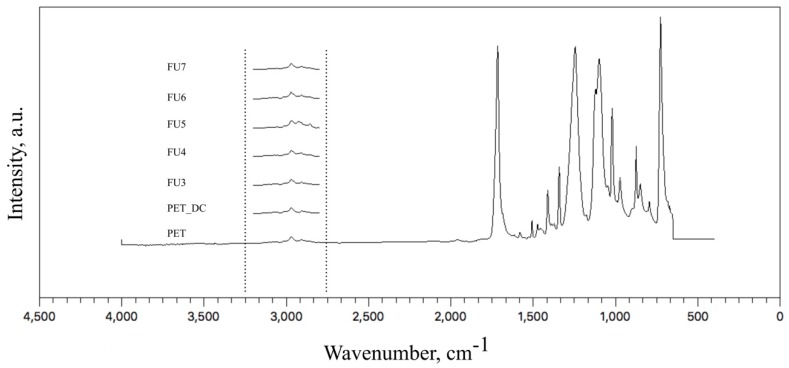
Attenuated total reflectance (ATR)-FTIR spectrum collected from the samples.

The chemical changes in the near-surface area of the samples were observed by attenuated total reflectance Fourier transform infrared spectra (ATR-FTIR) for the mid-IR range of 4000–400 cm^−1^, and the collected data are shown in Figure 4. The major peaks of PET were observed at the wavenumbers of: 720 cm^−1^ related to CH_2_ rocking vibrations; 795 cm^−1^ in the plane vibration of C–H; 850 cm^−1^ and 872 cm^-1^ corresponding to wagging vibration of O–CH_2_ and out-of-plane C–H vibration, respectively; 975 cm^−1^ due to stretching of O–CH_2_; 1015 cm^−1^ related to in-plane C–H vibration; 1095 cm^−1^ and 1243 cm^−1^ corresponding to asymmetric stretching vibration of C–O; 1340 cm^−1^ referring to CH_2_ wagging; 1410 cm^−1^ related to C–H bending vibration; 1471cm^−1^ related to CH_2_ bending; 1511 cm^−1^ referring to C–H bending vibration; 1718 cm^−1^ due to C=O stretching band [48,49,50]. The absorption peaks observed between 2850 and 3000 cm^−1^ were attributed to aliphatic C–H stretching vibrations [47,50].

Due to the penetration depth of ATR crystals of ZnSe (0.6 µm), which was well above the thickness of the coated FU (assumed to be nanometer size), the collected data were mostly belongs to the PET itself. Nevertheless, between wavenumbers 3000 and 2850 cm^−1^, especially for the FU5 samples, a slight intensity increase of peaks was observed, which refers to the immobilization of FU. The peak corresponds to the OH group vibration from the monosaccharide monomer. The peak at pH 5 showed the highest obtained intensity due to the highest amount of immobilized FU, as is discussed later on.

### 3.4. Surface Elemental Analysis by XPS 

Surface chemical compositions of the samples were revealed by using X-ray photoelectron spectroscopy (XPS), and results are listed in Table 2. The XPS spectra of the reference PET sample consisted only of carbon and oxygen content with the levels of 69.7% and 30.3%, respectively. After DC plasma treatment, the oxygen level increased to 40.9% due to the oxidative group functionalities introduced by plasma, and the carbon level decreased proportionally. Likewise, nitrogen was detected after plasma treatment with a level of 0.7%, due to the amine groups introduced by the applied plasma. Oxygen and nitrogen levels decreased for all fucoidan-immobilized samples as an indicator of fucoidan bonding. Nevertheless, sulphur content was lowest for the FU3 sample and highest for the FU5 sample, which signifies fucoidan immobilization. The highest sulphur content was observed at pH 5. Therefore, maximum anticoagulation activity is expected for the FU5 counterpart, according to the XPS data.

### 3.5. Blood Coagulation Activity 

The anticoagulation activity of the samples was examined to reveal prothrombin time (PT), activated partial thromboplastin time (aPTT) and thrombin time (TT). Therefore, three pathways of the blood coagulation cascade of the surface-mediated intrinsic pathway, the tissue-mediated extrinsic pathway and the common coagulation pathway were investigated in vitro and the results are given in Table 3.

**Figure 5:**
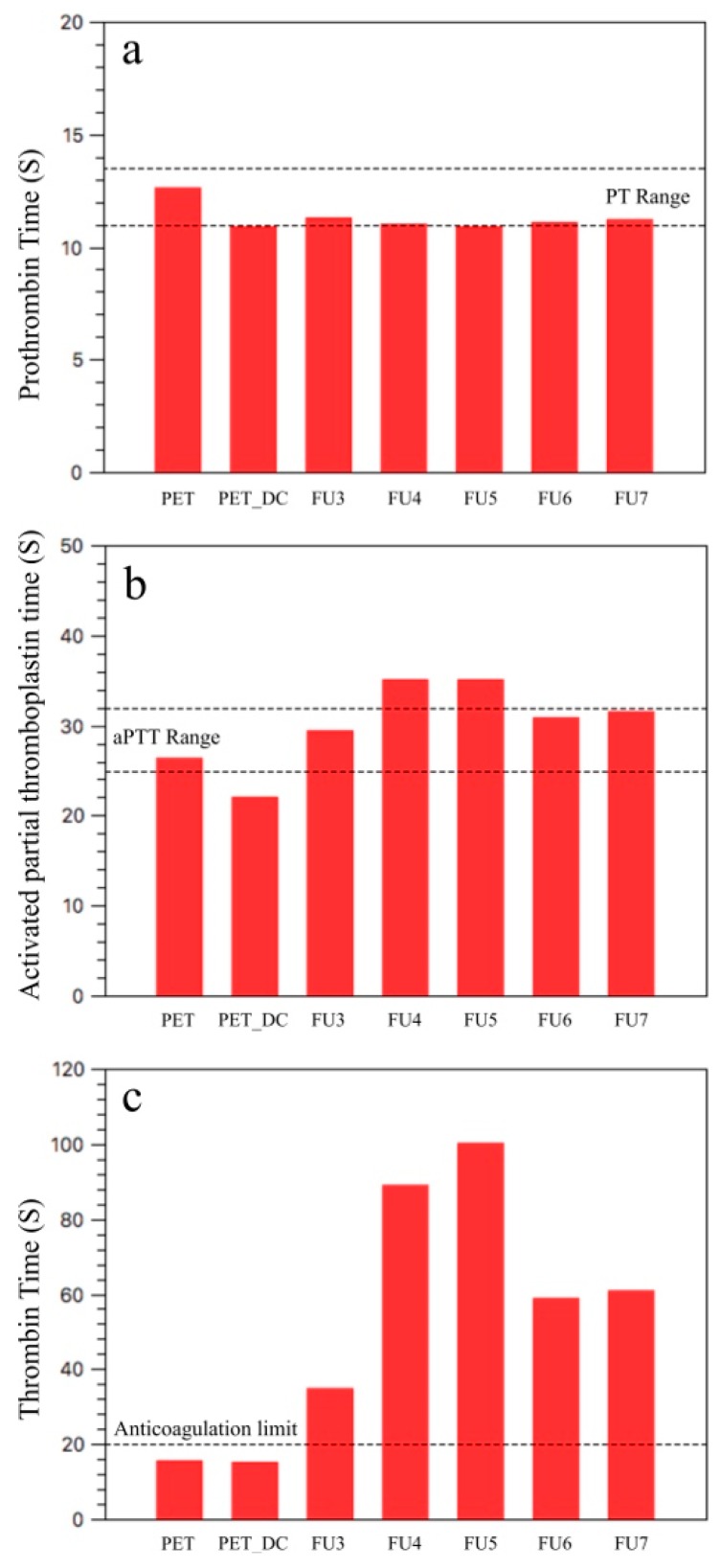
Anticoagulant activity results: (**a**) PT: prothrombin time (s); (**b**) aPTT: activated partial thromboplastin time (s); (**c**) TT: thrombin time (s).

PT measures the clot formation time in the extrinsic pathway, and the common coagulation pathway and PT range for the healthy donors is between 11 and 13.5 s [51]. According to the results of the PT assay, all samples are in the range with minor alteration (Figure 5a).

aPTT measures the coagulation activity of the intrinsic pathway and the common coagulation pathway, in contrast to PT. For the healthy donors, the time range of aPTT is between 25 and 32 s [52]. aPTT assay revealed that the PET surface is in the range by 26.3 s, but after DC plasma treatment it slightly decreased below the threshold by 22 s (Figure 5b). This is probably due to the increased surface roughness (which increases the hydrophilicity) that increases the fraction between the material surface and floating blood. The nitrogen content of PET_DC also needs to be taken into consideration. Fucoidan-immobilized samples FU3, FU6 and FU7 were within this range. By means of FU4 and FU5 samples, both were above the range by 35.1 s, which retarded the coagulation even further than that of upper anticoagulant limit of 32 s. 

TT measures the thrombin formation time by means of the conversion rate of fibrinogen to fibrin in the common coagulation pathway. The threshold for the anticoagulation activity is 20 s, which means a TT over 20 s is considered to be anticoagulant (Figure 5c) [52,53,54].

The reference PET sample and its plasma-treated counterpart were found to have TT values of 15.5 and 15.2 s, implicating that neither the PET nor the PET_DC surfaces influenced the common coagulation pathway to retard blood thrombus formation. All fucoidan-immobilized samples had a TT value over 20 s, starting from 34.7 s of the FU3 sample. The TT value was increased by pH to 88.7 s and was >100 s for FU4 and FU5 samples, respectively. The further increase of pH to 6 and 7, for samples FU6 and FU7, meant the TT value was decreased to 58.6 and 60.7 s, respectively. Nevertheless, it was well above the threshold of 20 s, which implies that the FU species were successfully immobilized onto the surface at each pH value from 3 to 7 and that all of them exhibited anticoagulant activity. Hence, the pH effect on FU immobilization onto the plasma-treated PET surface and its anticoagulation performance can be seen from the TT results.

As it is also seen in Figure 3e, the FU species on the surface were denser and more homogenous, so that XPS collected the highest sulphur content of 1.8 for the FU5 sample, which increased the heparin cofactor II (HCII)-mediated antithrombotic activity [30,36]. The sulphur content of 1.6 was observed for the FU4 sample, which was the second highest among all the samples that refer to sufficient FU immobilization, and its anticoagulant activity showed the second highest performance. Even if all the FU-immobilized samples at different pH values can be considered as an anticoagulant, for the long-term usage of blood-contacting device, it could be considered that the FU5 sample is the most suitable since its TT value exceeds 100 s.

## 4. Conclusions

In order to achieve anticoagulant activity, a polyethylene terephthalate (PET) surface, for use in blood-contacting devices, was treated by DC air plasma and fucoidan from *Fucus vesiculosus* (FU) and was immobilized in different pH values with a range of 3–7. The FU immobilization onto the PET surface after plasma treatment was found to be best at pH 5, which it also correlated with FTIR, SEM and XPS results, and it exhibited the highest anticoagulant activity, more than 100 s, which indicates that it is a fully anticoagulant FU-immobilized sample suitable for blood-contacting PET devices. Nevertheless, FU immobilizations were successful for all samples and exhibited anticoagulant activity well above that of the TT threshold of 20 s.

## Figures and Tables

**Table 1:** Water contact angle values of the samples.

Samples	Contact Angle (°)
PET	70.6 ± 0.60
PET_DC	22.09 ± 1.32
FU3	43.21 ± 2.67
FU4	39.08 ± 1.74
FU5	42.61 ± 2.92
FU6	39.74 ± 5.52
FU7	42.54 ± 7.45

**Table 2:** Collected elemental compositions in atomic percentages of the samples by X-ray photoelectron spectroscopy (XPS).

Samples	C1s	O1s	N1s	S2p	O1s/C1s	N1s/C1s	S2p/C1s
PET	69.7	30.3	0	0	0.435	0	0
PET_DC	58.4	40.9	0.7	0	0.700	0.012	0
FU3	63.4	35.7	0	0.2	0.563	0	0.003
FU4	63.7	34.5	0.2	1.6	0.542	0.003	0.025
FU5	63.6	34.4	0.2	1.8	0.541	0.003	0.028
FU6	63.7	35.7	0.1	0.5	0.560	0.002	0.008
FU7	63.8	35.6	0.1	0.5	0.558	0.002	0.008

**Table 3:** Anticoagulation activity results; PT: prothrombin time (s); aPTT: activated partial thromboplastin time (s); TT: thrombin time (s).

Samples	PT	aPTT	TT
PET	12.6	26.3	15.5
PET_DC	10.9	22	15.2
FU3	11.3	29.4	34.7
FU4	11	35.1	88.7
FU5	10.9	35.1	100+
FU6	11.1	30.8	58.6
FU7	11.2	31.5	60.7
